# The comparative burden of salmonellosis in the European Union member states, associated and candidate countries

**DOI:** 10.1186/1471-2458-6-4

**Published:** 2006-01-10

**Authors:** Birgitta de Jong, Karl Ekdahl

**Affiliations:** 1 Department of Epidemiology, Swedish Institute for Infectious Disease Control (SMI), Solna, Sweden; 2 Department of Medical Epidemiology and Biostatistics, Karolinska Institute, Stockholm, Sweden; 3 European Centre for Disease Prevention and Control (ECDC), Stockholm, Sweden

## Abstract

**Background:**

Salmonella is an infectious agents causing numerous cases of illness each year, and thereby having significant economic impact. Using returning Swedish travellers we estimated the burden of salmonellosis in different European countries.

**Methods:**

From the Swedish database on notifiable communicable diseases 15,864 cases with travel-associated salmonellosis acquired in Europe from 1997–2003 were retrieved. These cases were compared to a dataset from the same years on 14,171 randomly selected Swedish residents, with a history of recent overnight travel in Europe. Distribution of salmonellosis in returning travellers and the distribution of *Salmonella *Enteritidis was analysed for different member states in the European Union, associated and candidate countries. The risk of being notified with a salmonella infection after return from each European country/region was calculated, and compared with official reporting data rom these countries. Using Norway as reference country, we could 1) construct comparable incidence estimates and 2) calculate the "under-reporting" in each country compared to Norway.

**Results:**

The highest burden of salmonellosis was estimated for Bulgaria (2741/100,000), followed by Turkey with 2344/100 000 and Malta with 2141/100 000. *S*. Enteritidis is the dominating serotype, 66.9 % of all cases and phage type 4 accounts for 37.5 % of the *S*. Enteritidis cases

**Conclusion:**

Using returning tourists as a sentinel population can provide a useful base for comparison of disease burdens in different countries/regions. Focusing prevention of salmonellosis to prevention of egg and poultry associated S. Enteritidis infection will have a major impact from a public health perspective and will significantly lower the burden of disease in most European countries.

## Background

Salmonellosis is an important public health problem causing substantial morbidity, and thus also having a significant economic impact. Although most infections cause mild to moderate self-limited disease, serious infections leading to death do occur. In the United States it is estimated that 1.4 million non-typhoidal Salmonella infections with 400 deaths occurs annually [1]. Calculations from England and Wales regarding year 1995 resulted in an estimate of 102,227 indigenous cases, with 3,412 hospital admissions and 268 deaths [2].

The infective dose is usually high, but the bacteria grow well in most foodstuff. In food with a high fat content, e.g. chocolate and cheese, the infective dose is very low, and just a few bacteria may be sufficient to cause infection [3,4]. The susceptibility to infection varies; in infants, elderly, or compromised hosts, the critical infective dose is lower [5]. The onset of disease is often acute with diarrhoea, nausea and vomiting [6]. The incubation period is 1–3 (range <1–10) days. The carrier state is normally 4–6 weeks, but a few percent may be asymptomatic carriers for months or even years [3,7]. No vaccine is available against non-typhoidal salmonellosis. Using the Kauffmann-White scheme for antisera reaction to different bacterial O and H antigens, more than 2,500 *Salmonella *serovars have been identified, with prevalence patterns varying between different parts of the world [8,9]. In Europe *S*. Enteritidis has been the most reported serovar since the middle of the 1980s [10]. In the developed world salmonellosis due to *S*. Enteritidis is most often associated with consumption of poultry and eggs [11,12].

The European Union (EU) consists of 25 member states along with 4 associated EEA/ EFTA (European Economic Area/ European Free Trade Area) countries and 4 candidate countries, with altogether 630 millions inhabitants. The recent establishment of the European Centre for Disease Prevention and Control (ECDC), shows that control and prevention of communicable diseases are high on the EU political agenda. The *Salmonella *species is one of the eight micro-organisms in the EU Zoonoses Monitoring Directive (2003/99/EG), which is mandatory to always monitor [13]. The subject matter and scope of this directive is to ensure that zoonoses are properly monitored and that foodborne outbreaks receive proper epidemiological investigation, to enable the collection in the EU of the relevant information necessary to evaluate trends and sources. In the Zoonosis Control Regulation (2160/2003) which regulates the control of salmonella and other specified foodborne zoonotic agents, salmonella is the only agent specified [14]. This directive and regulations shows that salmonellosis is a disease considered to have a high impact on the human health in the EU. However, public health actions need to be based on best possible surveillance data, but the ability to detect and report enteric zoonoses varies considerably between the European countries, hampering meaningful comparisons between countries [15].

In this study we are using Swedish notification data on all cases of salmonellosis, who acquired the infection during travel in Europe, and a unique database on travel patterns to give an estimate of the comparative burden of non-typhoidal salmonellosis in different European countries.

## Methods

### Notification data on salmonellosis

The statutory notification of salmonellosis in Sweden is regulated in the Communicable Disease Act. All notifications are submitted to the Swedish Institute for Infectious Disease Control (SMI), both by the physician having seen the patient (clinical notification) and by the laboratory having diagnosed the causative agent (laboratory notification). The clinical notifications include information of epidemiological relevance, e.g. route of infection, risk group, and likely place and country of infection. The laboratory notifications include the *Salmonella *serovar and if a *S*. Enteritidis or *S*. Typhimurium, phage types are included. The clinical and laboratory reports for each patient are merged, using a unique personal identification number. The personal identification number is used in all contacts with the health care, both private and public.

For this study, notification data for the period January 1997 to December 2003 were used. All records in the national surveillance database concerning patients notified with salmonellosi*s *were retrieved. The clinical notification has the information in which country the patient has acquired the infection and this information was used to judge if the patient was a case of travel associated salmonellosis or not. Patients with stated indigenously acquired infection (i.e. infected in Sweden) or patients for which information on likely country of infection was either missing or "unknown" were excluded. Also patients with a travel history outside Europe were excluded. Furthermore, the dataset was "cleaned" from infections in recently arrived immigrants, refugees and non-Swedish visitors (identified through the personal identification number).

### Travel patterns

As denominator on travel patterns, a commercial database, the Tourist Database (TDB) was used [16]. This dataset is based on a randomized selection of 2,000 persons of the Swedish population every month. These persons are telephone interviewed with questions on both business and pleasure travel during last month, and the data weighted and extrapolated to give an estimate of the total number of Swedish travellers to different destinations. Out of the total database, containing data on almost 170,000 interviews, altogether 14,171 records from respondents with a history of recent overnight travel in Europe were extracted. These 14,171records represents all persons in the TBD, which has been travelling in Europe. Data on country/region of travel was used for analysis. No data on any illness is available from this dataset.

### Serovar typing and phage typing

Serovar typing of the *Salmonella *strains was performed by agglutination using antiserum from Reagensia, Stockholm, Sweden. Interpretation of serovars was made according to the latest edition of the Kauffmann-White scheme from the WHO International Reference Laboratory [8]. Phage typing of *S*. Enteritidis was performed according to the Colindale scheme [17]. These results were included in the laboratory notification from the Swedish national salmonella reference laboratory.

### Statistical methods

The risk of disease per 100,000 travellers was calculated using salmonellosis notification data from the actual country/region as numerator and the estimated total numbers of travellers from the TDB as denominator. Data for each country on the reported number of salmonellosis cases were retrieved from WHO Surveillance Program for Control of Foodborne Infections and Intoxications in Europe, 8^th ^report, year 2000, if not other source is stated. An under-detection index was calculated with Norway as the reference country (the country with the lowest risk of salmonellosis per 100 000 Swedish travellers, and a good national reporting system). The under-detection index is calculated by dividing the incidence/100 000 inhabitants in the country with the risk/100 000 Swedish travellers who visited the country and this quotient is then dived with the quotient from the reference country (Norway). This index denotes estimated number of salmonellosis cases not notified for every notified case. To measure the burden of salmonellosis in each country this index was multiplied with the reported incidence from the actual country. The method, with a detailed discussion on strengths and limitations has been previously published [15].

### Ethical considerations

The TDB contains aggregated data only. Notification data is regulated by the Communicable Disease Act, and contain full personal identification. The subset of the notification database extracted for this study does not contain any information that could be linked to a specific person. The Ethical Committee of the Karolinska Institute, Stockholm, Sweden, approved the study.

## Results

### Travel pattern

Data from the 14,171 respondents in the TDB with recent overnight travel to different European countries/regions during the study period 1997–2003 were weighted and projected to give an estimate of the number of journeys to each European country/region. According to these estimates, Swedish travellers did approximately 60 million overnight journeys to other European countries (78% leisure trips and 22% business trips) during the period.

### Salmonellosis in returning travellers

For the study period 1997–2003, altogether 15,864 patients were notified with a salmonella infection after a journey in Europe and out of them 10,607 had an infection caused by *S*. Enteritidis. No cases of salmonellosis were reported among travellers from Luxembourg and Liechtenstein.

The total risk of being notified with salmonellosis was 26.2/100,000 travellers. The lowest risk was seen after a journey to Norway (0.2/100,000 travellers) followed by Finland (0.4/100,000 travellers) and Norway was hence used as reference. The highest risks was observed in travellers returning from Bulgaria (129/100,000 travellers), Turkey (110/100,000 travellers and Malta (101/100,000 travellers) (Table 1).

The total risk of being notified with a salmonella infection caused by *S*. Enteritidis, all phage types, was 17.5/100,000 travellers. The highest risks were seen for traveling in Bulgaria with 87.5/ 100,000 travellers, Portugal 66.4/100,000 travellers, and Turkey 53/100,000 travellers, data not shown.

### Disease burden

When using our data to estimate the actual incidence of salmonellosis, the incidence varied from 4/100,000 inhabitants in Norway up to 2,741/100,000 inhabitants in Bulgaria (Table 1). Countries with the lowest burden of disease were situated in the northern parts of Europe while countries with a higher burden were situated in the southern parts of Europe. Poland is the only country in the northern part that had a high burden of disease. Countries in the eastern parts of Europe also tended to have a higher burden than the countries situated in the western parts of Europe.

### Serovar distribution

**Figure 1:**
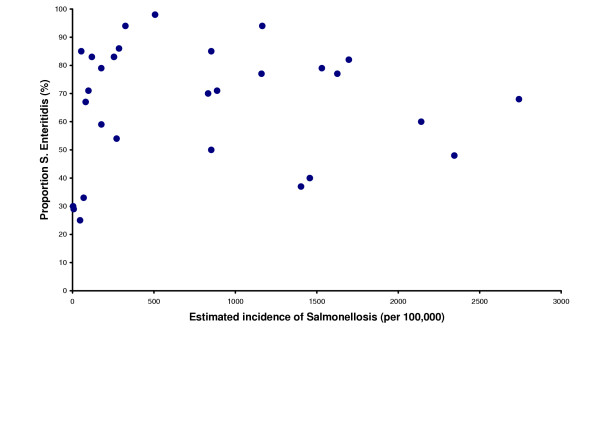
**Proportion of *Salmonella *Enteritidis in returning travellers, plotted against the estimated incidence of salmonellosis. **Proportion of *Salmonella *Enteritidis in returning travellers, plotted against the estimated incidence of salmonellosis. Each dot represents one country in the study.

Details on the different serovars of *Salmonella*, clearly showed the relative high burden of *S*. Enteritidis in Europe, accounting for 67% of the cases. However, the proportion of *S*. Enteritidis cases from the different countries varied from 25% (Iceland) up to 98% (Latvia). The second most frequent serovar was *S*. Typhimurium accounting for 9% of the cases (Table 2). When plotting the proportion of *S*. Enteritidis in returning travellers against the estimated incidence no correlation could be detected (Figure 1).

### Phage type distribution of *S*. Enteritidis

Of the 10,607 cases of *S*. Enteritidis in our database, data on phage type (PT) was available for 10,479 (99%) cases. In all, 48 different phage types were represented among the notified cases during the study period. Regional variations in serovar distribution could be noted. *S*. Enteritidis PT 4 is the most dominating phage type, accounting for 35% of all *S*. Enteritidis cases, while PT 1 accounts for 22%. On the Iberian Peninsula and in some eastern parts of Europe PT 1 is the dominating phage type, while PT 14b is dominating in Greece and PT 8 in Czech Republic, Denmark and Slovakia, (Table 3). Spain is the only European country having persons returning with PT 44 (28 cases). An outbreak with PT 34 in Denmark caused 73 cases and this phage type is also reported from Spanish travellers.

### Duration of stay

In the TBD the duration of stay is measured in number of night spent abroad. Figure 4 shows the percentage of night spent in each country. There are differences in the duration of stay, which is correlated to the distance to each country and the accessibility. The duration of stay is not available in the notification from the physician seeing the case of salmonellosis.

## Discussion

To measure the burden of non-typhoidal salmonellosis in different countries using the reported cases in each country will not produce comparable numbers, since the reporting systems in the different European countries are very disparate. In some countries all cases are reported, while in some other countries just the hospitalized cases. Furthermore, in many countries indigenous and imported cases are not considered separately.

To get comparable data, we used a different approach, basing our estimates on returning travellers to Sweden. Reliable denominator data on travel are essential when using travel-related disease data for calculations of risks [18]. The TDB database gives information on each country/region visited with at least one overnight stay (with information obtained directly from the travellers), providing us with a unique opportunity to relate surveillance data to a better-suited travel denominator when calculating the burden of salmonellosis in European countries. This database has previously been used for estimating the risk of travel-related diseases in various parts of the world [9,19-23], and the strengths and limitations of using this approach has been discussed in detail in these articles.

As the numerator we used the notifications in the national surveillance system. Since Sweden has a uniform national system for reporting infectious diseases and the notification database rests on both clinical and laboratory notifications, the sensitivity of the notification system is comparatively good. Using the capture-recapture technique to estimate the proportion of cases being reported by either clinicians or laboratories, more than 99 percent of all diagnosed *Salmonella *infections were found to be reported each year in 1998 to 2002 [24]. Even with a good notification system, the reported cases likely only represent the tip of the iceberg. Salmonellosis is an acute, mostly self-limited disease, and most cases never come under the attention of a physician, and even less are microbiologically investigated. This however, should not in any major way affect how the countries in this study compare to each other.

The data presented in this study are based on Swedish travellers and their behaviours and risks could never be fully representative for those of the native population in the respective countries. The risk estimates are also biased towards the parts of the countries with most tourists. However, since the data concerning the different countries are based on the same methods, we believe the risks estimates for the countries from this study are more suited for comparisons than the reported national incidence figures from the notification systems – with different structures and quality. Furthermore, a study from the UK estimated the incidence of salmonellosis presenting to general practitioners in 1995 to 157 per 100,000 and the incidence of laboratory confirmed cases to 80 per 100,000 [25], figures in the same magnitude as our estimate of 119 per 100,000 in that country.

In this study certain countries in Europe stood out as having a comparatively very high burden of non-typhoidal salmonellosis. A north-south gradient could be clearly distinguished, with increasing incidences the more south in Europe the country is situated. Also a west-east gradient is shown with a higher burden in eastern countries/regions. Being the most common serovar in most regions of the world, *S*. Enteritidis is especially dominating in Europe, where almost 67% of all salmonellosis were due to this serovar. In Africa, Asia and America, this serovar has been less dominating and the variety of circulating serovars greater [9].

The duration of travel may affect the risk per 100,000 travellers since persons going to southern countries are mostly on a leisure trip with a longer duration compared to trips to neighbouring countries and thereby the risk for these southern countries could be overestimated. In this study the 4 countries with the highest burden of non-typhoidal salmonellosis were tourist destinations where Swedish travellers mostly stay 1–2 weeks. However, the country with the fifth highest burden, Poland, had travel durations similar to other neighbouring countries of Sweden. This indicates that our method is useful, but improvements can be introduced if duration of stay for the infected traveller is obtainable.

The sometimes very large differences between the estimated disease burden from this study, and the reported figures from the single countries are problematic from public health point of view, therefore the strategy on the future European-level surveillance presented by the ECDC in October emphasized the needs for more comparable surveillance data,.

Due to very rigorous control methods in the agricultural area, Sweden has an extremely low domestic incidence of salmonellosis in humans and well as in animals [26,27]; less than 0.1 % of food-producing animals are found to carry *Salmonella *[26]. This favourable situation is shared with the EU member state Finland and the associated countries Iceland and Norway. These four countries have also managed to keep free from the pandemic of egg-associated *S*. Enteritidis that is contributing to a great extent to the burden of salmonellosis in other European countries. At least 75% of all notified episodes of salmonellosis in Sweden are travel-related. Even in neighbouring Denmark, the situation is the reversed, with only 25% of the most common serovar, *S*. Enteritidis being related to travel [28]. When becoming members of the EU, Sweden together with Finland received specific *Salmonella *guarantees, which states that fresh beef, veal and pig meat together with poultry meat should be tested for the presence of Salmonella bacteria before importation. If any serovar of *Salmonella *is found, importation is not allowed. These guarantees have contributed to the maintenance of the excellent Salmonella situation in these member states and other states may apply for similar guarantees when they have approved control programmes in the agricultural area. Our study shows that these guarantees have a positive effect on public health, resulting in a very low level of indigenous cases of salmonellosis.

The two most common serovars, *S*. Enteritidis and *S*. Typhimurium, are well known to have different animals as reservoir for the disease in Europe. *S*. Enteritidis is shown to be the most frequent serovar isolated from poultry and poultry products including table eggs, while *S*. Typhimurium is the most frequent serovar in pigs, pork, beef and veal. In cattle S. Dublin is the most frequent isolated serovar but S. Typhimurium is the next frequent serovar. [29]. However, *S*. Typhimurium is also rather common in poultry and *S*. Enteritidis is isolated from beef and pork.

## Conclusion

Our data shows that focusing prevention of salmonellosis to prevention of *S*. Enteritidis infection will have a major impact from a public health perspective and will significantly lower the burden of salmonellosis in most European countries. *S*. Enteritidis is well known to originate from poultry products and products prepared with raw shell eggs. Aiming at reducing the presence of *S*. Enteritidis in poultry products and eggs will have a positive side-effect since not only *S*. Enteritidis will be reduced but also other serotypes of Salmonella present in these products resulting in an even lower burden of salmonellosis in Europe. However, since there was no direct correlation between the proportion of *S*. Enteritidis and the estimated overall incidence of salmonellosis, also other actions than those directed against infected poultry and eggs are needed to get down the burden of salmonellosis in those countries with the highest incidence. The new ECDC has identified zoonoses as one of its main priorities, and will together with the EU Member States, the European Commission and the European Food Safety Agency (EFSA) play a pivotal role in the future actions against salmonellosis in Europe.

## Competing interests

The author(s) declare that they have no competing interests.

## Authors' contributions

Both authors participated in the design of the study, performed the calculations and drafted the manuscript. Both authors has read and approved the final manuscript.

## Pre-publication history

The pre-publication history for this paper can be accessed here:



## Figures and Tables

**Table 1:** Comparison of risk of Salmonella infection in Swedish travellers to national reported incidence and estimated incidence of salmonellosis.

**Country**	**Risk per 100,000 travellers (95% CI)**	**Percent S. Enteritidis among Swedish travellers**	**Percent S. Typhimurium among Swedish travellers**	**Reported incidence per 100,000 inhabitant**	**Under-detection index**	**Estimated incidence per 100,000 inhabitant**
Norway	0.2 (0.11–0.38)	30	40	3.77	1.0 (ref.)	4
Finland	0.4 (0.28–0.58)	29	39	13.1	0.6	8
Iceland	2.2 (0.79–6.1)	25	75	129	0.4	47
Estonia^1^	2.6 (0.94–7.2)	85	2	40.6	1.3	54
Ireland	3.2 (1.8–3.8)	33	25	16.0	4.3	69
Denmark	3.8 (3.4–4.3	67	15	44.4	1.8	81
Netherlands^2^	4.6 (3.2–6.6)	71	29	12.6	7.7	98
United Kingdom	5.6 (4.8–6.5)	83	7	27.9	4.3	119
France	8.4 (7.2–9.7)	59	13	21.5	8.3	178
Germany	8.3 (7.4–9.3)	79	13	96.1	1.8	177
Austria	12.0 (9.7–14.9)	83	2	85.6	3.0	255
Italy	12.8 (11.2–14.6)	54	18	20.6	13.1	271
Belgium	13.5 (10.0–18.2)	86	10	153	1.9	286
Lithuania^1^	15.3 (4.5–52.5)	94	0	32.5	10.0	325
Latvia^1^	23.9 (12.4–46.5)	98	1	43.4	11.7	507
Greece	39.2 (36.4–42.2)	70	8	8.5	97.7	833
Croatia	40.1 (32.7–49.1)	85	8	27.9	30.6	852
Slovenia	40.1 (32.7–49.1)	50	20	85.1	10.0	852
Hungary	41.8 (33.7–51.8)	71	34	163	5.5	888
Czech Republic	54.8 (45.5–66.1)	77	5	391	3.0	1161
Slovakia	54.8 (45.5–66.1)	94	0	336	3.5	1165
Cyprus	66.0 (56.2–77.6)	37	15	19.7	71.2	1403
Romania	68.6 (39.5–119)	40	31	4.4	332	1457
Spain^3^	72.0 (68.5–75.7)	79	7	14.9	103	1531
Poland	76.5 (65.5–89.4)	77	3	100	16.2	1626
Portugal	79.9 (68.0–93.9)	82	7	4.49	378	1697
Malta	101 (69.1–147)	60	13	23.1	92.6	2141
Turkey	110 (97.2–125)	48	4	39.2	59.8	2344
Bulgaria	129 (103–162)	68	17	10.1	271	2741

^1 ^Risk per 100,000 travellers calculated on figures from 2002–2003

^2 ^Incidence calculated on 63% of inhabitants since reported number of cases is from laboratories with a catchments area of 63% of inhabitants

**Table 2:** The number per country of the most common Salmonella serovars from notified cases in returning Swedish travellers with non-typhoidal salmonellosis 1997–2003.

	**Salmonella serovars**										
**Country**	**Agona**	**Blockley**	**Enteritidis**	**Hadar**	**Heidelberg**	**Infantis**	**Montevideo**	**Newport**	**Saintpaul**	**Typhimurium**	**Virchow**
Norway			3							4	
Finland			8			1			1	11	1
Denmark	2		239	4	1	15	2	3	1	53	4
Iceland			1							3	
Belgium		1	60	2						7	
United Kingdom	1		173	1						4	1
France	1	3	149	5	2	2		5	2	32	9
Ireland			4							3	
Netherlands			25							10	
Switzerland			7								
Germany		1	323	3	1	7	1		1	53	5
Austria		1	101	1				1		2	
Italy		3	184	1	4	5		3	4	60	2
Malta	5	2	78	4		7				17	3
Portugal	1	4	498	11	2			4		42	4
Spain	14	3	4,891	100	16	17	16	34	6	446	78
Greece	8	31	1,315	39	11	23	19	21	2	156	36
Baltic countries			143	1		1				1	
Bulgaria	10		350	5	3	3	4		1	90	
Poland	1		510	49		4	1	2	4	18	4
Romania			19	2						15	
Czech Rep. + Slovakia	5		290	1	1	8			4	16	
Hungary	2	7	164	16		5			14	8	5
Cyprus	2	80	202	26	10	17	3	1	1	81	20
Turkey	94	6	674	17	1	6	17	4		58	210
**Total**	**147**	**143**	**10,637**	**289**	**52**	**121**	**63**	**79**	**41**	**1,203**	**383**

**Table 3:** The number per country of the most common Salmonella Enteritidis phage types in returning Swedish travellers with non-typhoidal salmonellosis 1997–2003.

	**Phage types**								
**Country**	**1**	**4**	**6**	**6a**	**6b**	**8**	**13a**	**14b**	**21**
Norway						2			
Finland	5					2			1
Denmark	6	23	52	2		61	2		7
Iceland		1							
Belgium	2	34		5				6	10
United Kingdom	10	110	14	6		5	4	3	3
France	8	75	12	8	1	18			3
Ireland		4							
Netherlands		18		2	1			1	2
Switzerland		5				2			
Germany	10	196	25	9		37	1	4	20
Austria	1	46	4			35	4	1	2
Italy	24	75	3	2		24	2	20	14
Malta		18		58				1	1
Portugal	201	96	8	46	2			39	25
Spain	1,689	1,612	128	211	11	86	20	76	151
Greece	139	328	4	120	36	24	3	380	73
Baltic countries	77	20	7			2			9
Bulgaria	7	191	2	2	1	26	15	39	36
Poland	43	183	65	2	1	101		1	84
Rumania	2	9	1			1		1	1
Czech Republic + Slovakia	6	33	12	1		206	5	2	11
Hungary	4	73	30			34	1	5	1
Cyprus	79	71	2	2		1		7	1
Turkey	20	377	73	8		3		2	52
**Total**	**2,263**	**3,713**	**450**	**489**	**54**	**694**	**69**	**588**	**519**

**Table 4:** Duration of travel shown as percentage of nights spent in each country

	**Number of nights**												
**Country**	**1**	**2**	**3**	**4**	**5**	**6**	**7**	**8**	**9**	**10**	**11–17**	**18–24**	**>24**
Norway	23	20	14	14	6	7	5	1	2	1	4	1	1
Finland	35	25	9	5	4	3	4	2	1	2	5	2	1
Iceland	0	6	26	8	15	8	7	4	9	0	12	5	1
Estonia	14	44	13	5	4	1	8	2	2	2	3	1	1
Ireland	6	9	17	15	12	18	11	3	0	3	5	1	1
Denmark	31	30	15	7	5	4	4	1	1	1	2	1	1
Netherlands	21	23	16	15	5	6	1	2	3	1	5	2	1
United Kingdom	8	17	21	16	9	7	6	3	2	2	6	2	2
France	6	7	9	9	7	11	14	6	1	5	16	4	3
Germany	21	27	19	9	6	5	3	2	1	1	5	1	1
Austria	3	3	9	5	10	12	21	8	6	7	11	2	2
Italy	1	12	11	6	5	12	13	5	3	4	20	4	3
Belgium	26	18	17	15	5	4	8	3	1	1	2	1	1
Lithuania	2	25	35	18	13	4	0	0	0	2	0	0	2
Latvia	2	26	24	21	17	2	3	1	1	1	2	0	0
Greece	0	1	1	1	2	23	38	2	1	1	29	2	1
Slovenia	0	4	8	0	4	0	0	4	0	0	42	15	23
Hungary	1	11	16	15	11	5	7	3	1	3	10	8	9
Czech Republic	1	9	27	26	12	10	6	2	1	3	2	0	0
Slovakia	14	21	0	0	0	0	7	7	0	7	0	0	43
Cyprus	0	0	0	0	2	21	49	1	1	0	24	1	2
Romania	4	4	5	4	5	22	29	4	1	2	17	1	2
Spain	1	2	3	3	2	20	32	3	2	2	23	3	3
Poland	16	27	13	9	8	2	7	2	3	3	6	1	1
Portugal	5	2	6	4	6	23	29	4	0	2	17	1	2
Malta	0	0	6	3	6	25	28	3	2	0	5	22	2
Turkey	0	0	4	3	1	21	30	2	0	1	33	3	2
Bulgaria	0	0	6	1	0	22	40	5	0	0	25	1	2
